# The role of web sharing, species recognition and host-plant defence in interspecific competition between two herbivorous mite species

**DOI:** 10.1007/s10493-016-0079-5

**Published:** 2016-08-09

**Authors:** Yukie Sato, Juan M. Alba, Martijn Egas, Maurice W. Sabelis

**Affiliations:** 1 Institute for Biodiversity and Ecosystem Dynamics, University of Amsterdam, P.O. Box 94240, 1090GE Amsterdam, Netherlands; 2 Sugadaira Montane Research Center, University of Tsukuba, Ueda, Nagano 386-2204 Japan

**Keywords:** Aggregation, Exotic species, Endemic species, Intra- and interspecific web sharing, Direct plant defence

## Abstract

When competing with indigenous species, invasive species face a problem, because they typically start with a few colonizers. Evidently, some species succeeded, begging an answer to the question how they invade. Here, we investigate how the invasive spider mite *Tetranychus evansi* interacts with the indigenous species *T. urticae* when sharing the solanaceous host plant tomato: do they choose to live together or to avoid each other’s colonies? Both species spin protective, silken webs on the leaf surfaces, under which they live in groups of con- and possibly heterospecifics. In Spain, *T.* *evansi* invaded the non-crop field where native *Tetranychus* species including *T. urticae* dominated. Moreover, *T. evansi* outcompetes *T. urticae* when released together on a tomato plant. However, molecular plant studies suggest that *T. urticae* benefits from the local down-regulation of tomato plant defences by *T. evansi*, whereas *T. evansi* suffers from the induction of these defences by *T. urticae*. Therefore, we hypothesize that *T. evansi* avoids leaves infested with *T. urticae* whereas *T. urticae* prefers leaves infested by *T. evansi*. Using wild-type tomato and a mutant lacking jasmonate-mediated anti-herbivore defences, we tested the hypothesis and found that *T. evansi* avoided sharing webs with *T. urticae* in favour of a web with conspecifics, whereas *T. urticae* more frequently chose to share webs with *T. evansi* than with conspecifics. Also, *T. evansi* shows higher aggregation on a tomato plant than *T. urticae*, irrespective of whether the mites occur on the plant together or not.

## Introduction

Understanding how invasive species overcome the initial hurdles of establishing in a new habitat and ultimately spread, is essential to understand why some species successfully invade and others do not (Hulme 2006). Specifically, much attention has been paid to genetic and physiological traits of invasive species, because colonization of a new environment typically starts from a small number of founders (Holway and Suarez 1999). Tolerance to reduced genetic diversity and adaptation to a new abiotic environment are deemed important aspects to initiate new populations (e.g. Sakai et al. 2001). Besides these traits, however, recent research focuses on behavioural traits associated with heterospecific competition as one of the key traits of invasive species (e.g., Holway and Suarez 1999; Chapple et al. 2012). For example, biotype B of the whitefly, *Bemisia tabaci* (Gennadius), recently invaded and displaced indigenous biotypes in Zhejiang, China and Queensland, Australia, and the invasion success is partly explained by difference in mating behaviour between biotypes: when they are mixed, biotype B increases the number of copulations to ensure female acquisition of conspecific sperm whereas indigenous biotypes reduce the number of copulation by interference of biotype B (Liu et al. 2007). The invasive fire ant, *Solenopsis invicta* Buren, has displaced native ants all over the world, and the larger group size of its multiple-queened colonies is thought to be key to outcompete indigenous ants (Holway and Suarez 1999). These behavioural traits can be key for invasive species, but more generally speaking, such behavioural traits are important to understand how newcomers compete with incumbent occupants.

Here, we focus on the invasive spider mite *Tetranychus evansi* Baker and Pritchard (Acari: Tetranychidae) infesting solanaceous plants. This species originates from South America, but has become invasive first in Africa and then in Europe (Boubou et al. 2012). Nowadays, the mite is an important pest of tomato plants (*Solanum lycopersicum* L.) in Africa and parts of Europe, but not in South America, its area of origin (Navajas et al. 2012). During the invasion process, *T. evansi* interacted with *Tetranychus urticae* Koch, as *T. urticae* is endemic in these geographic regions (Helle and Sabelis 1985; Navajas et al. 2012). Moreover, *T. urticae* is also an important pest of tomato plants, and both species are found together on solanaceous crops in greenhouses and in the field (e.g., Ferrero et al. 2011; Ferragut et al. 2013). In Spain, *T.* *evansi* invaded a non-crop field where native *Tetranychus* species including *T. urticae* dominated (Ferragut et al. 2013). Moreover, *T. evansi* outcompeted *T. urticae* when released together on a tomato plant (Sarmento et al. 2011b). In sharp contrast with these field observations, molecular studies show that co-occurrence on tomato plants may benefit *T. urticae*, but comes at a cost to *T. evansi*. Many strains of *T. urticae* induce jasmonate (JA)-mediated and salicylate (SA)-mediated plant defences in tomato (Li et al. 2002; Kant et al. 2004, 2008; Ament et al. 2004; Sarmento et al. 2011a) whereas *T. evansi* down-regulates these defences (Sarmento et al. 2011a; Alba et al. 2015). JA-mediated defences reduce female fecundity of *T. urticae* (Li et al. 2002; Ament et al. 2004; Kant et al. 2008), and prior infestation of the plants by *T. urticae* reduces female fecundity of *T. evansi* (Sarmento et al. 2011a, b). Hence, it is predicted that *T. evansi* may have a strategy to outweigh the competitive disadvantage against *T. urticae*.

One possible mechanism that *T. evansi* may employ to prevent *T. urticae* from taking advantage of the suppressed plant defences, is covering the exploited leaf area with such a dense web that *T. urticae* cannot penetrate it (Sarmento et al. 2011b). Both species spin protective, silken webs on the leaf surface of their host plant, and the mites feed, mate, reproduce and develop under these webs. Webs produced by *T. evansi* are denser compared to those of *T. urticae*, and these dense webs do reduce leaf access by *T. urticae* (Sarmento et al. 2011b). In addition, *T. evansi* produces even more web when it experiences *T. urticae* nearby (Sarmento et al. 2011b). Even when *T. urticae* penetrates the dense web, other mechanisms may work as a barrier against exploitation by *T. urticae*. For example, males of *T. evansi* prefer to mate with *T. urticae* females instead of conspecific females, whereas *T. urticae* males prefer to mate with conspecific females, indicating that *T. evansi* interferes with the reproduction of *T. urticae* (Sato et al. 2014, 2016). The cost of heterospecific mating for females is estimated to be small, since *T. urticae* females can produce female offspring by mating with conspecific males after the heterospecific mating (Sato, personal observation) and male offspring production is similar between heterospecifically mated females and virgin females suggesting that heterospecific mating does not result in aborted offspring (Clemente et al. 2016). However, competition experiments on a tomato plant showed that reproductive interference does affect population dynamics (Sato et al. 2014). Hence, the dense web and the reproductive interference may work in concert after *T. evansi* colonies have sufficiently developed. However, it might be less effective when the colony size of *T. evansi* is still small or when *T. evansi* is establishing new colonies, because the amount of silk is strongly correlated with the number of female spider mites (Le Goff et al. 2010), and because reproductive interference is frequency-dependent (Kuno 1992). Therefore, in the early phase of colonization there may be another mechanism preventing exploitation of *T. evansi* by *T. urticae*.

In this paper, we investigate web sharing behaviour of *T. evansi* and *T. urticae* to better understand their interaction in the early phase of colony establishment. Founder females should make a decision in which places and with whom they establish colonies. Spider mites in the genus *Tetranychu*s live in groups, and it is known that heterospecific web sharing easily occurs because of the function of webs as shelters against predators (Yano 2012). However, *T. evansi* should avoid sharing webs with *T. urticae* and aggregate with conspecies to protect the profitable place against *T. urticae*. First, we investigated the web-sharing probabilities of *T. evansi* female pairs, *T. urticae* female pairs and heterospecific female pairs, after they were introduced on the same tomato leaflet. Second, we carried out a choice test by releasing one *T. evansi* or one *T. urticae* female on a tomato leaflet on which there were already two webs, one from *T. evansi* and one from *T. urticae*. Lastly, we released *T. evansi* and *T. urticae* on a tomato plant either separately or together, and recorded the distribution of mites when they reached the second to third generation. To reduce the effect of JA-mediated plant defence and also to investigate the effect of JA-mediated plant defence on mite behaviour, we used JA-deficient mutant tomato plants, *def*-*1*, besides wild-type tomato plants, in these experiments.

## Materials and methods

### Tomato plants

We used wild-type tomato plants (*S. lycopersicum* cv. Castlemart) and the JA biosynthetic mutant *def*-*1* tomato plants (*S. lycopersicum* cv. Castlemart background) (Howe et al. 1996). Tomato seeds were sown in 12-cm pots in a greenhouse and were allowed to grow for 3 weeks. Subsequently, plants were transferred to a climate chamber (25 °C; 60 % RH; 16:8 h light: dark photoperiod) for another week for experiments.

### Mites

We used *T. evansi* and *T. urticae* collected from *Solanum nigrum* L. in Málaga (N36°34′29″, W5°57′35″), Spain, August 2010. *Tetranychus evansi* was reared on detached wild-type tomato leaves, and *T. urticae* was reared on detached common bean leaves (*Phaseolus vulgaris* L.) on wet cotton wool in a plastic box under constant climatic conditions (25 °C; 60 % RH; 16:8 h light: dark photoperiod) at the University of Amsterdam, the Netherlands. At least 1 month before experiments, a part of the *T. urticae* colony was moved to detached tomato leaves under the same conditions.

### Web sharing

To investigate whether *T. evansi* and *T. urticae* share web, we introduced two females from the same or different species on a detached leaflet of wild-type tomato plants (*S. lycopersicum* cv. Castlemart), and measured the frequency of the two females sharing a web. To investigate whether JA-mediated plant defences have an effect on the probability of web sharing, we also performed the experiment using leaflets from *def*-*1* tomato plants. *Tetranychus urticae* benefits from web sharing with *T. evansi* because the latter down-regulates plant defences even on detached leaf discs (Sarmento et al. 2011a, b), but web sharing with *T. evansi* brings also the risk of reproductive interference by *T. evansi* (Sato et al. 2014). The risk of reproductive interference would be much higher in virgin females than in mated females, because females of spider mites generally show strong first-male sperm precedence and secondary matings are less effective (Boudreaux 1963; Helle 1967). Therefore, it was expected that virgin females might share a web with individuals of their own species to avoid reproductive interference, whereas mated females may show less preference. We therefore used both virgin and mated females. The experimental design is summarized in Table 1.

**Table 1:**
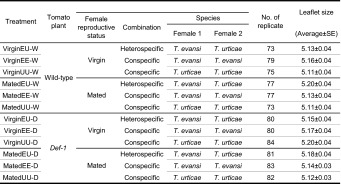
Experimental design of web sharing in *Tetranychus evansi* pairs, *T. urticae* pairs and the heterospecific pairs

For preparation of virgin and mated females, we used leaf discs (1.5 cm diameter) that were punched from detached tomato leaves. The leaf discs were placed on wet cotton wool spread in plastic trays. Females in the teleiochrysalis stage (moulting stage preceding adult phase) were collected from each colony, and each placed on a separate leaf disc. The stage of the females was checked 24 h later, and the females that moulted into adults were used as virgin females in the experiment. To prepare mated females, teleiochrysalis females were introduced on a leaf disc together with two males collected from the same mite colony. The stage of the females was checked 24 h later, and the females that moulted into the adult phase were used as mated females.

The size of the leaflets on which the mites are released was expected to affect the probability of web sharing; we therefore measured the length of leaflets, and used leaflets of 4.5–6.0 cm long. Two virgin or mated females were released together on a leaflet that was placed on wet cotton wool spread in a plastic tray. One day after introduction of the females, pairs were scored as web sharing when the two females were within one web or as non-sharing when the two females were apparently in separate webs, as determined by visual inspection of webbing through a binocular microscope. When the separation of webs was unclear, we counted the pairs as sharing. Pairs were not included in the analysis when either of the females was walking outside the web on the leaflet or on the wet cotton wool during the observation (157 of 1392 pairs).

To compare the probability of web sharing in conspecific pairs between *T. evansi* and *T. urticae*, we constructed a generalized linear mixed model (GLMM; *glmer* in the package *lme4* from the statistical package R) using data in which the combination of females were conspecific (VirginEE-W, VirginUU-W, MatedEE-W, MatedUU-W, VirginEE-D, VirginUU-D, MatedEE-D and MatedUU-D in Table 1). The dependent variable was binomial, with a value of 1 when females shared a web and 0 if not, and the explanatory variables were plant genotype (wild type or *def*-*1* mutant), mite species (*T. evansi* or *T. urticae*) and female reproductive status (virgin or mated). We incorporated leaflet size in the model as a random factor. The effect of each explanatory variable on the dependent variable was tested by comparing the models with and without the explanatory variable using a likelihood ratio test. When significant effects were detected in the interactions, GLMMs were constructed for each mite species and for each female reproductive status, and the effects of each explanatory variable was tested in the same way.

To determine whether *T. evansi* females shared webs with *T. urticae* females, we constructed a GLMM using the data in which one of two females was *T. evansi* (VirginEU-W, VirginEE-W, MatedEU-W, MatedEE-W, VirginEU-D, VirginEE-D, MatedEU-D, and MatedEE-D in Table 1). To determine whether *T. urticae* females share webs with *T. evansi* females, we constructed a GLMM using the data in which one of two females was *T. urticae* (VirginEU-W, VirginUU-W, MatedEU-W, MatedUU-W, VirginEU-D, VirginUU-D, MatedEU-D and MatedUU-D in Table 1). In each model, the dependent variable was binomial, with a value of 1 when females shared a web and 0 if not, and the explanatory variables were plant genotype, female reproductive status, combination of females (hetero- or conspecific pairs) and their interactions. We incorporated leaflet size as a random factor in the models. The effects of each explanatory variable on the dependent variable was tested in the same way as the comparison in conspecific pairs between *T. evansi* and *T. urticae*. We used statistical package R (v.2.14.2) for the analyses (R Development Core Team 2012).

### Choice test

To investigate the preference of females of *T. evansi* and *T. urticae* for webs constructed by conspecific or heterospecific females, we allowed females to choose between a web made by one *T. urticae* female and a web made by one *T. evansi* female using *def*-*1* leaflets. We prepared virgin females in the same manner described above and used them in this experiment. Since the mites possibly have a preference for certain locations on the leaflet, we created leaflets that varied web location: with *T. evansi* web near the tip of the leaflet and *T. urticae* web on the basal part of the leaflet or vice versa, as follows.

The surface of the leaflets was divided into the basal part and the upper part by placing a string (ca. 2 mm diameter and 4 cm long) made of wet cotton wool. Subsequently, one *T. evansi* and one *T. urticae* were released on either part respectively. One day later, their establishment was checked and the cotton wool string was removed. After the leaflet surface had dried up, one *T. evansi* or one *T. urticae* female was released in the middle of the leaflet. The location of the web and the position of the three females were checked after 24 h. If two conspecific females were together in the web made by the former conspecific occupant and one heterospecific female was alone, we scored the newcomer as preferring the conspecific web and female, and vice versa. If the three females were each in a separate web, we scored it as the newcomer having constructed a new web. If the three females were together in one of the two webs, we judged the newcomer’s choice by the location. We never found that there were three webs and three females were together. The length of leaflets was measured and we used leaflets of 6.0-8.6 cm long. The leaflets were placed on wet cotton wool spread in plastic trays.

We compared the preference for conspecific web between *T. evansi* and *T. urticae.* Triples were not included when any female was walking outside web or on the wet cotton wool during the observation (3 triples/87 triples). To determine whether *T. evansi* and *T. urticae* preferred conspecific web rather than heterospecific web, we constructed a GLMM with a binomial error distribution, in which the dependent variable was the proportion of choice of conspecific webs, and the explanatory variables were species of newcomer (*T. evansi* or *T. urticae*), location of conspecific former occupant (basal or upper) and the interaction as fixed factors and leaflet size as a random factor. To analyse the proportion of females that constructed a web instead of using existing webs, we constructed a GLMM for the proportion that females constructed a web by themselves in the same way with the previous model. The effect of each explanatory variable on the dependent variable was tested in the same way as the comparison in the previous experiments. We used R (v.2.14.2) for the analyses (R Development Core Team 2012).

### Aggregation on a tomato plant

To investigate aggregation patterns of *T. evansi* and *T. urticae* and to investigate the effect of the presence of heterospecifics on their gregarious behaviour, we used the records of the distribution of the two species on a *def-1* tomato plant which was infested with *T. evansi*, *T. urticae* or both species in the interspecific competition experiment in Sato et al. (2014). We introduced four virgin or mated females and four virgin males (2 days old) on the same leaflet of a tomato plant as founders. In the treatment for the mixture, two males and two females of both species were released. To ensure their establishment, we checked the number of mites for 3 days after mite introduction, and replaced missing mites, thereby reducing the probability that small initial differences in numbers were magnified through exponential growth. After releasing the mites on the plant, the adult females of each species were counted and the positions recorded once per week over a period of 4 weeks.

As a measure of aggregation, we calculated the index of mean crowding, $m^{*} = \left( {\frac{{\mathop \sum \nolimits_{j = 1}^{Q} x_{j}^{2} }}{{\mathop \sum \nolimits_{j = 1}^{Q} x_{j} }} - 1} \right)$, where *Q* is the total number of leaflets of the tomato plant and $x_{j}$ is the number of individuals on the *j* th leaflet (*j* = 1, 2, 3,···, *Q*) (Lloyd 1967). We also calculated the mean numbers of female mites per leaflet (hereafter, mean density: *m*). We constructed a linear mixed model of the index of mean crowding (m*) with species (*T. evansi* or *T. urticae*), treatment (single species or mixture), female reproductive status (virgin or mated) and the interactions as fixed effects, and with tomato plant and weeks as random effects because of repeated measurements (*lmer* in the package *lme4* and *lmerTest* from the statistical package R). Because the index of mean crowding (m*) depends on the mean density (*m*) (they show a linear relation) (Iwao 1968), we added mean densities of the mites and the interactions as fixed effects. To select a subset of explanatory variables from a larger set (model selection), we performed automatic backward elimination of the saturated model (*step* in the package *lmerTest* from R). Then, we checked the effects of each fixed effects on the model using the final model. We used R (v.2.14.2) for the analyses (R Development Core Team 2012).

## Results

### Web sharing

**Fig. 1:**
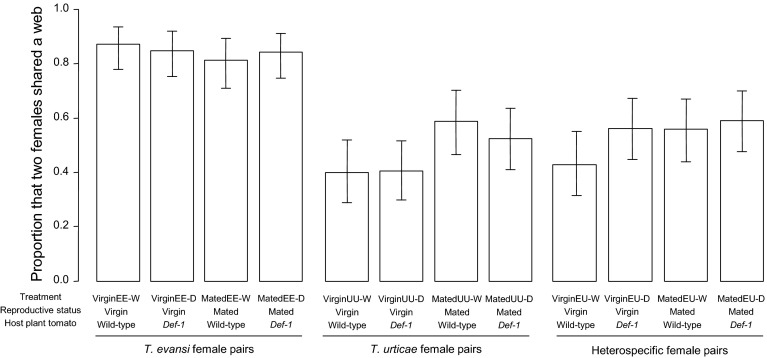
The probability of con- and heterospecific web sharing in *Tetranychus evansi* and *T. urticae* on wild-type and *def*-*1* tomato plants. *Error bars* indicate 95 % confidence intervals of the probabilities. The number of replicates and the details of treatments are shown in Table 1

In the model for comparison of web-sharing probability in conspecific pairs between *T. evansi* and *T. urticae*, the effect of female reproductive status on the probability that two conspecific females shared a web, differed between *T. evansi* and *T. urticae* (species × female reproductive status: likelihood ratio test, *χ* ^2^ = 5.444, df = 1, *p* = 0.02; Fig. 1). In *T. evansi*, approximately 80 % of the conspecific pairs shared a web regardless of their reproductive status (likelihood ratio test, *χ* ^2^ = 0.817, df = 1, *p* = 0.37) and regardless of plant genotype (likelihood ratio test, *χ* ^2^ = 0.034, df = 1, *p* = 0.86). In *T. urticae*, the probability of web sharing was significantly higher in mated female pairs than virgin female pairs (likelihood ratio test, *χ* ^2^ = 7.335, df = 1, *p* = 0.007), regardless of plant genotype (likelihood ratio test, *χ* ^2^ = 0.28, df = 1, *p* = 0.597). In the comparisons between species for each female reproductive status, *T. evansi* pairs always showed higher probabilities of web sharing than *T. urticae* pairs (virgin female pairs: likelihood ratio test, *χ* ^2^ = 76.421, df = 1, *p* < 0.001; mated female pairs: likelihood ratio test, *χ* ^2^ = 27.564, df = 1, *p* < 0.001). Plant genotype did not have a significant effect on the probability of web sharing in either female reproductive status (virgin females: likelihood ratio test, *χ* ^2^ = 0.048, df = 1, *p* = 0.83; mated females: likelihood ratio test, *χ* ^2^ = 0.062, df = 1, *p* = 0.80).

Next, we analysed whether *T. evansi* females shared webs with *T. urticae* females as with conspecific females. The probability that a female *T. evansi* shared a web with a female *T. urticae* was significantly lower than the probability of conspecific web sharing (likelihood ratio test, *χ* ^2^ = 71.926, df = 1, *p* < 0.001): 40–60 % of heterospecific pairs shared a web whereas approximately 80 % of the conspecific pairs shared a web (Fig. 1). Female reproductive status and plant genotype did not have significant effects on the probability (female reproductive status: likelihood ratio test, *χ* ^2^ = 0.353, df = 1, *p* = 0.55; tomato plant: likelihood ratio test, *χ* ^2^ = 1.690, df = 1, *p* = 0.19).

Then, we analysed whether *T. urticae* females shared webs with *T. evansi* females as with conspecific females. In *T. urticae*, the probability of web sharing was significantly lower in virgin females than mated females (Fig. 1; likelihood ratio test, *χ* ^2^ = 8.476, df = 1, *p* = 0.004). The probability of web sharing seemed to be higher in heterospecific pairs than conspecific pairs, but the difference was not significant (likelihood ratio test, *χ* ^2^ = 2.127, df = 1, *p* = 0.15). The effect of plant genotype was not significant (likelihood ratio test, *χ* ^2^ = 0.490, df = 1, *p* = 0.48).

### Choice test

**Fig. 2:**
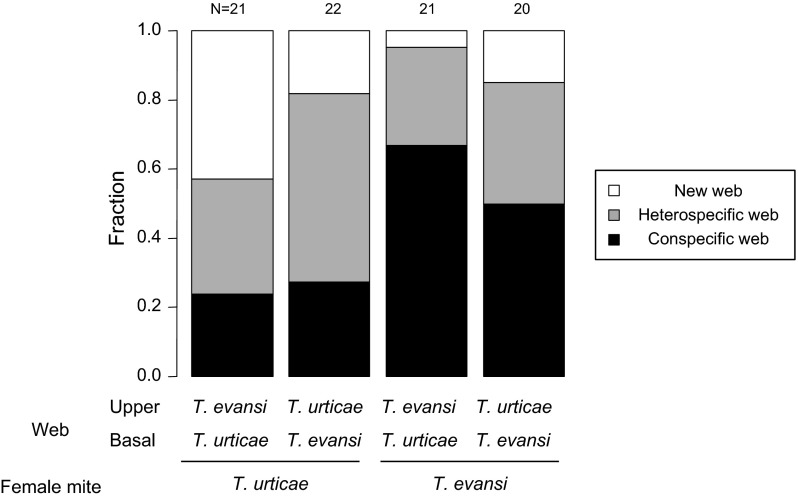
Preference of *Tetranychus evansi* females and of *T. urticae* females for webs constructed by *T. evansi* or *T. urticae*. The location of the web is a two-level treatment: in one *T. evansi* web was on the *upper part* and *T. urticae* web was on the *basal part* of a tomato leaflet and in the other it is vice versa. Some of the females did not choose one of the two webs present, but constructed a web by themselves. *Numbers* above *bars* indicate the number of replicates (N)

The proportion of females choosing conspecific webs was different between *T. evansi* and *T. urticae* (Fig. 2; likelihood ratio test, *χ* ^2^ = 5.224, df = 1, *p* = 0.022). For both species, females preferred *T. evansi* web, although some females constructed new webs by themselves (Fig. 2). The effect of location of webs was not significant (likelihood ratio test, *χ* ^2^ = 1.130, df = 1, *p* = 0.29). The proportion of females that constructed new webs instead of choosing existing webs was significantly higher in *T. urticae* than *T. evansi* (likelihood ratio test, *χ* ^2^ = 5.990, df = 1, *p* = 0.014). The proportion of females that constructed webs instead of choosing existing webs was higher when the location of conspecific webs was basal part of the leaflet (likelihood ratio test, *χ* ^2^ = 4.423, df = 1, *p* = 0.035).

### Aggregation on a tomato plant

**Fig. 3:**
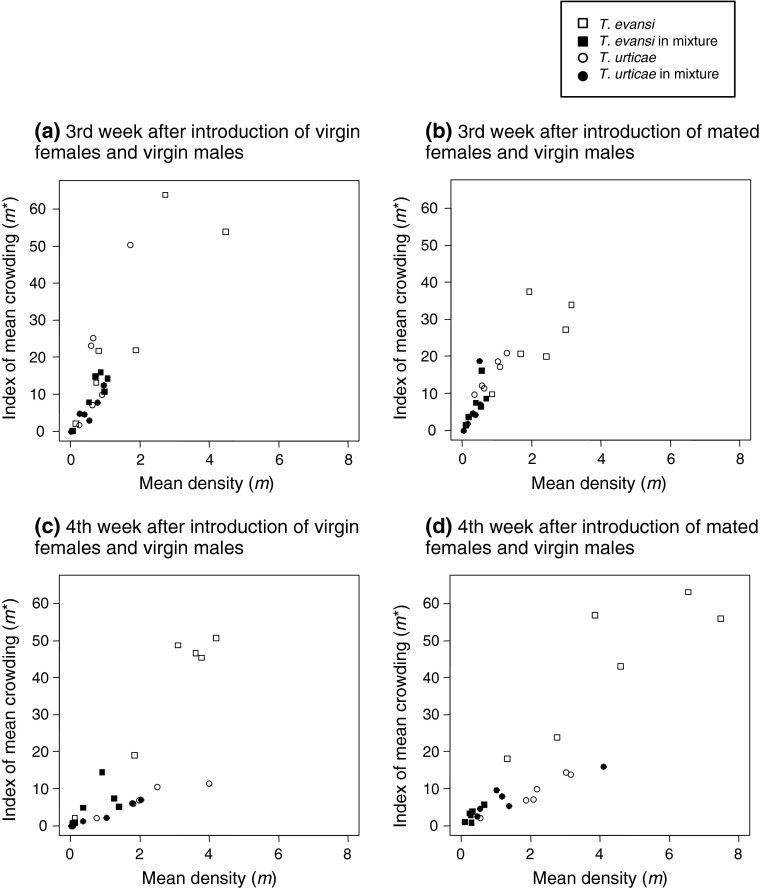
Relationship between m* (index of mean crowding) and *m* (mean density; the average number of female mites per leaflet) in *Tetranychus evansi* and *T. urticae.* *Open squares* and *circles* show m* of *T. evansi* and *T. urticae* females in which each species was introduced separately. *Filled squares* and *circles* show m* of *T. evansi* and *T. urticae* females on a tomato plant in which both species were introduced together

In the analyses of the mean crowding (m*), we used the data of third and fourth week after releasing the mites (i.e., the second to third generation of descendants from the released mites), because the number of females in the first to two weeks were too small for reliable analyses (Sato et al. 2014). Results of model selection (Table 2) and the final model (Table 3) show that the regression coefficient of m* along mean density (*m*) was significantly higher in *T. evansi* than *T. urticae* (Fig. 3). This indicates that *T. evansi* shows higher gregariousness than *T. urticae*. However, treatment (single species or mixture) did not have a significant effect on the slope of m* along *m* (Tables 2, 3; Fig. 3), indicating that both species females did not change their aggregation pattern depending on the presence of the other species. The slope was significantly steeper in the populations in which founder females were virgin than the populations in which founder females were mated (Tables 2, 3; Fig. 3).

**Table 2:** Results of backward elimination of non-significant effects of saturated linear mixed model of the index of mean crowding (m*) for the fixed effects (a) and the random effects (b)

Fixed effects	df	denDF	*F*	*p*
(a)				
Mean density (m)	1	89.0	88.02	<0.001
Treatment	1	88.4	7.18	0.009
Species	1	88.1	0.57	0.45
Female reproductive status	1	88.0	1.51	0.22
Mean density (m) × treatment	1	85.1	0.16	0.69
Mean density (m) × species	1	88.6	26.21	<0.001
Treatment × species	1	84.0	0.003	0.95
Mean density (m) × female reproductive status	1	88.0	8.95	0.004
Treatmemt × female reproductive status	1	82.0	0.15	0.70
Species × female reproductive status	1	87.0	0.79	0.38
Mean density (m) × treatment × species	1	83.1	1.31	0.26
Mean density (m) × treatment × female reproductive status	1	80.1	0.07	0.80
Mean density (m) × species × female reproductive status	1	86.0	2.31	0.13
Treatment × species × female reproductive status	1	81.0	0.39	0.53
Mean density (m) × treatment × species × female reproductive status	1	79.0	0.40	0.53

Random effects	df	χ^2^	*p*
(b)			
Week	1	9.290	0.002
Tomato plant	1	0.910	0.34

The *p* values for the fixed effects are calculated from *F* test based on Sattethwaite’s approximation. The *p* values for the random effects are based on likelihood ratio test

**Table 3:** Final model detected by backward elimination of non-significant effects of linear mixed model of the index of mean crowding (m*)

Fixed effects	Estimate	SE	*t*	*p*
Intercept	2.23	3.41	0.66	0.60
Mean density (m)	8.55	0.77	11.15	<0.001
Treatment	4.64	1.73	2.68	0.009
Mixture versus single species				
Species	1.60	2.13	0.75	0.45
*T. evansi* versus *T. urticae*				
Female reproductive status	−2.53	2.06	−1.23	0.22
Mated versus virgin				
Mean density (m) × species	−6.34	1.24	−5.12	<0.001
Mean density (m) × female reproductive status	3.22	1.08	2.99	0.004

Random effects	Variance	SD
Week	17.23	4.15
Intercept		
Residual	50.86	7.13

N = 96 from 3rd and 4th weeks after mite introduction

## Discussion

Here, we studied whether *T. evansi* females and *T. urticae* females display behaviour that promotes living together in webs or not. *Tetranychus urticae* females shared webs with *T. evansi* females more frequently than with conspecific females. Furthermore, in the choice tests, *T. urticae* females chose webs of *T. evansi* females more than those of conspecific females. However, *T. evansi* females shared webs with *T. urticae* females less frequently than with conspecific females, and preferred webs of their own species to those of *T. urticae* females. In addition, *T. evansi* females showed higher gregariousness than *T. urticae* females, although neither species changed its aggregation pattern depending on the presence of heterospecifics. These results meet our prediction: *T. evansi* females avoid sharing webs with *T. urticae* females and aggregate with conspecies to protect the profitable place against *T. urticae* because of local induction of plant defences caused by *T. urticae* infestation.

Given that *T. evansi* females avoid living together with *T. urticae* females, the question arises: how do they discriminate *T. urticae* from its own species? One possibility is that *T. evansi* uses plant defences as signals of presence of *T. urticae* on the host plant, because *T. evansi* down-regulates the plant defence whereas *T. urticae* induces the plant defences (Li et al. 2002; Kant et al. 2004, 2008; Ament et al. 2004; Sarmento et al. 2011a; Alba et al. 2015). We did not observe apparent differences in the probability of heterospecific web sharing between wild-type (cv. Castlemart) and *def*-*1* tomato plants which are deficient in mounting JA-mediated plant defences. Our results may not be able to completely reject the hypothesis on the role of plant defences in web sharing, because *T. urticae* also induce salicylic acid-related defences (Kant et al. 2004; Sarmento et al. 2011a; Alba et al. 2015). This pathway is induced in *def*-*1* plants, and probably enhanced due to the absence of the negative crosstalk between JA and SA. However, SA defences have a relatively low impact on mite performance (Villarroel et al. 2016). The other possibility is that *T. evansi* uses silk and faeces produced by *T. urticae* as a signal of presence of *T. urticae.* Clotuche et al. (2014) investigated chemical cues affecting gregarious behaviour of *T. urticae,* and found that extracts of silk with black faeces (but not with white faeces and eggs) using hexane or methanol are attractive for *T. urticae* individuals. Use of volatile chemical cues from their own faeces was found in several spider mites. For example, the social spider mite, *Stigmaeopsis miscanthi,* uses the chemical volatiles contained in its faeces to detect defaecation sites (Sato et al. 2003). The use of chemical cues from silk and faeces would be worth testing in *T. evansi*. As well as chemical cues from silk and faeces, they possibly use sex pheromones to discriminate *T. urticae* from its own species. In our previous study, we observed that males of *T. evansi* prefer to copulate with females of *T. urticae* rather than conspecific females (Sato et al. 2014, 2016), suggesting that the compounds or concentration of sex pheromones is different between *T. urticae* and *T. evansi* females. Sex pheromones from *T. urticae* females are possibly able to work as attractant for *T. evansi* males but repellent for *T. evansi* females at the same time. Further research is necessary to determine which mechanisms *T. evansi* uses to detect the presence of *T. urticae*.

*Tetranychus urticae* showed a preference for sharing webs with *T. evansi.* It can be explained by their relationship via host plants, because *T. evansi* makes infested host plant leaves more suitable by down-regulating plant defences (Sarmento et al. 2011a, b; Alba et al. 2015). However, sharing webs with *T. evansi* also brings costs: *T. urticae* is subject to reproductive interference from *T. evansi* (Sato et al. 2014) and a dense web of *T. evansi* hampers feeding activity of *T. urticae* (Sarmento et al. 2011b). In addition, we found that virgin *T. urticae* females more frequently construct their own webs instead of using existing webs. This behaviour can be regarded as an avoidance of reproductive interference, because virgin females are much more vulnerable to reproductive interference than mated females in spider mites. A few papers reported heterospecific aggregation in other species (Hodge and Storfer-Isser 1997; Krams and Krama 2002; Briones-Fourzán et al. 2008) including the spider mites *T. urticae* and *T. kanzawai* (Yano 2012). In the case of *T. urticae* and *T. kanzawai*, it is suggested that they share webs, because the web of both species serves as protection from predatory mites and the effect of protection from predatory mites exceeds the costs of heterospecific web sharing (Yano 2012). Considering the difference in web sharing behaviours between virgin and mated *T. urticae* females, future studies should investigate the balance of benefits and costs of heterospecific web sharing in different sexes, stages and circumstances.
